# Assessing Vegetation Canopy Growth Variations in Northeast China

**DOI:** 10.3390/plants14010143

**Published:** 2025-01-06

**Authors:** Lijie Lu, Lingxue Yu, Xuan Li, Li Gao, Lun Bao, Xinyue Chang, Xiaohong Gao, Zhongquan Cai

**Affiliations:** 1 State Key Laboratory of Black Soils Conservation and Utilization, Northeast Institute of Geography and Agroecology, Chinese Academy of Sciences, Changchun 130102, China; yx17365967150@163.com (L.L.); lixuan01@iga.ac.cn (X.L.); gaoli24@mails.ucas.ac.cn (L.G.); baolun21@mails.ucas.ac.cn (L.B.); changxinyue22@mails.ucas.ac.cn (X.C.); 15244604281@163.com (X.G.); zhongqcai@163.com (Z.C.); 2 Faculty of Computing, Harbin Institute of Technology, Harbin 150006, China; 3 National Key Laboratory of Smart Farm Technologies and Systems, Harbin 150006, China

**Keywords:** climate change, canopy development, canopy senescence, Northeast China, phenological changes

## Abstract

Studying climate change’s impact on vegetation canopy growth and senescence is significant for understanding and predicting vegetation dynamics. However, there is a lack of adequate research on canopy changes across the lifecycles of different vegetation types. Using GLASS LAI (leaf area index) data (2001–2020), we investigated canopy development (April–June), maturity (July–August), and senescence (September–October) rates in Northeast China, focusing on their responses to preseason climatic factors. We identified that early stages saw canopy development acceleration, with over 71% of areas experiencing such acceleration in April and May. As the vegetation grew, the accelerating canopy development slowed down, and the canopy reached its maturation earlier. By analyzing the partial correlation between canopy growth and preseason climatic factors, it was identified that changes in canopy growth were most significantly affected by preseason air temperature. A positive correlation was observed in the early stages, which shifted to a negative correlation during canopy maturation and senescence. Notably, the transition timing varied among different vegetation types, with grasslands (June) occurring earlier than forests (July) and farmlands (August). Additionally, grassland canopy growth showed a stronger response to precipitation than forests and farmlands, with a lagged effect of 2.50 months. Our findings improve understanding of vegetation canopy growth across different stages, holding significant importance for ecological environmental monitoring, land-use planning, and sustainable development.

## 1. Introduction

Over the past few decades, global vegetation greenness has generally increased. This is due to the combined effects of factors such as the fertilization effect of CO_2_ and climate warming [1,2,3]. The greening of the vegetation canopy can enhance ecosystem productivity. This could potentially become a major driving force of global terrestrial carbon sequestration. This increase in vegetation greenness is mainly observed in mid-to-high latitudes, particularly in regions undergoing afforestation and agricultural intensification, such as China and India [4,5,6,7]. Meanwhile, significant changes have occurred in plant phenology with climate warming. These changes result in an earlier spring leaf-out and a delayed autumn leaf fall, which in turn extend the growing season at both regional and global levels [8,9,10,11]. Plant phenology serves as one of the most sensitive biological indicators of climate change. Additionally, its variations have significant impacts on the water, carbon, and energy cycles between terrestrial ecosystems and the climate system [12,13,14].

Traditional vegetation phenology monitoring often relies on ground-based datasets [15,16]. Currently, several independent ground-based datasets are used to assess land surface phenology: (1) eddy flux tower measurements of Gross Primary Production [17]; (2) repeat landscape images collected through the PhenoCam Network [18]; (3) observations of plant phenology collected across the landscape [19]; (4) observations from national phenology networks [20]. The ground-based observational methods have the advantages of producing intuitive and accurate results, being simple and easy to implement, and allowing for high observation frequency [21]. The attributes of these data enable precise capture of temporal milestones in vegetation phenological changes and facilitate comparison of phenological differences among various species. However, this method is not suitable for large-scale phenology change analysis. Remote sensing has been widely used in the study of vegetation dynamics, which greatly expands the scope of traditional plant phenology observation due to its advantages of long-term and large-scale observation [22,23]. This method detects the timing of phenological events based on the distribution of time-series vegetation indices [8,24,25]. Currently, satellite-derived plant phenology indicators typically focus on the start of the growing season (SOS), end of the growing season (EOS), and length of the growing season (LOS) [9,26,27]. However, these indicators lack an evaluation of vegetation growth rate changes throughout the entire cycle from canopy development to senescence.

Furthermore, due to the natural regulation of growth rhythm, subsequent vegetation growth states also undergo linked changes as phenology advances. A greener spring may be followed by a greener summer (positive coupling, known as the “growth continuation effect”) [28]. Conversely, a greener spring could be followed by a browner summer (negative coupling, known as the “growth continuation effect”) [29]. Notably, for the Northern Hemisphere, recent studies have shown that between 2002 and 2022, the spring and summer vegetation growth shifted from a positive coupling to a negative coupling [30]. In addition, many scholars have studied changes in canopy greenness amplitude on monthly, seasonal, or annual scales. They identified that the overall speed of canopy development is increasing [31]. However, this increase does not imply that the rate of vegetation change is the same across different months. This is because there are differences in climate variation among different months [32]. Additionally, vegetation growth in different months/seasons is controlled by various climatic drivers [33,34]. To address this issue, some scholars have studied the overall speed of canopy development using the ratio of LAI (leaf area index) amplitude during the green-up period to the length of the green-up period [35]. Others have used monthly NDVI (Normalized Difference Vegetation Index) increments as an indicator to study the speed of canopy development and senescence [36,37].

Notably, the selected NDVI values and their interpretation can be affected by shadows, especially in forests with more complex canopy structures and more pronounced clustering [38]. Additionally, traditional vegetation indices are influenced by leaf senescence but not falling off, resulting in a certain lag in the inversion of the yellowing period [39]. LAI is defined as the ratio of the total leaf surface area of plants to the unit area of land. LAI can reflect the number of plant leaves, changes in canopy structure, plant community vitality, and their environmental effects. It overcomes the low sensitivity of NDVI in high vegetation areas and provides higher accuracy in vegetation phenology monitoring. The metric of vegetation LAI increment (VLAI) holds great potential for describing canopy development and senescence. A positive or negative value of monthly VLAI suggests canopy development or senescence, respectively, and the absolute value of the VLAI indicates the speed of canopy changes. Currently, some scholars have analyzed phenological changes based on LAI data [14,22,35]. However, few have used monthly LAI increments as an indicator to study the speed of canopy development, maturation, and senescence. Meanwhile, different plants adopt various phenological response strategies to climate change. Phenological variations within and between species differ in magnitude and direction [40,41]. However, there is limited exploration of differences in canopy development, maturation, and senescence rates among different vegetation types and their responses to climate change. Therefore, it is necessary to conduct more detailed vegetation phenology analyses.

The vegetation types in Northeast China are diverse and particularly sensitive to climate change, making it a natural testing ground for analyzing changes in vegetation phenology. Additionally, vegetation phenology in different regions varies in sensitivity to climate [8]. Further research is needed to explore the underlying driving mechanisms of climate change on vegetation canopy development and senescence rates. Therefore, in this study, we focus on grasslands, farmlands, and forests in Northeast China, using GLASS LAI data and climate data including air temperature (TEM), precipitation (PRE), and solar radiation (SRAD) from 2001 to 2020 to investigate changes in monthly canopy development, maturation, and senescence rates of vegetation and their response to climate change. This study specifically explores the following questions:

(1) What is the spatiotemporal distribution of monthly canopy development, maturation, and senescence rate changes under the background of climate change?
(2) What are the differences in canopy development, maturation, and senescence rate changes among different vegetation types?
(3) What are the response characteristics of canopy development, maturation, and senescence rate changes in different vegetation types to climate change?

## 2. Results

### 2.1. Trends in LAI and VLAI

**Figure 1:**
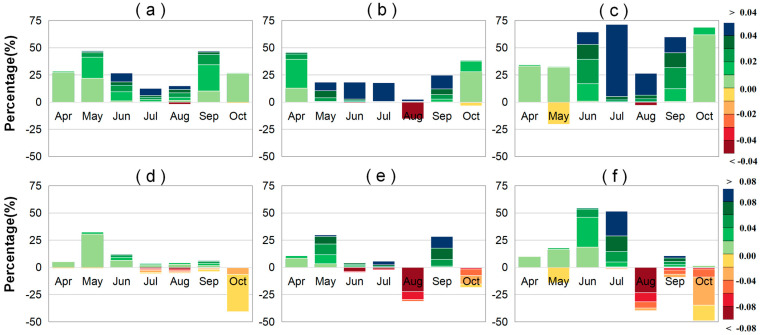
Stacked percentage chart of regions where the different trend (*p* < 0.05) ranges in monthly LAI (leaf area index) (or VLAI: monthly LAI increments) for grasslands (**a**,**d**), forests (**b**,**e**), and farmlands (**c**,**f**) in Northeast China from 2001 to 2020. The upward and downward bars represent percentages of significant positive and negative trends, respectively.

**Figure 2:**
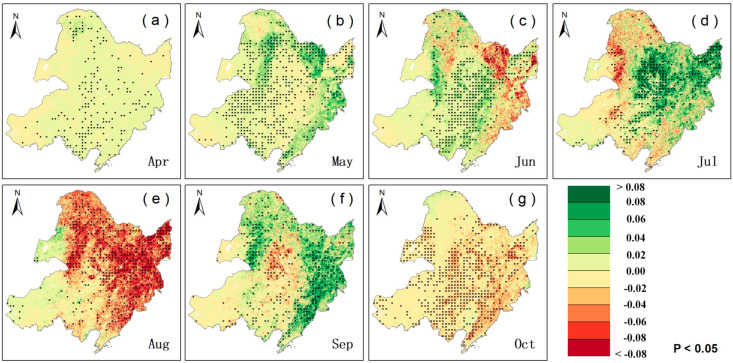
Spatial patterns of trends in monthly VLAI from 2001 to 2020 in Northeast China (with monthly designations: (**a**) = April, (**b**) = May, (**c**) = June, (**d**) = July, (**e**) = August, (**f**) = September, (**g**) = October. The regions labeled with black dots represent locations with a significant trend in VLAI (*p* < 0.05).

We employed the slope trend analysis method to determine the 20-year trends of LAI and VLAI. The trend of LAI helps us understand the overall growth status of vegetation. The trend of VLAI helps us understand the development status of the vegetation canopy. To better analyze the trends of LAI and VLAI, we categorized the magnitude of these changes. We used a stacked bar chart to illustrate the overall trend of these changes (Figure 1). A graded color map was employed to depict the spatial distribution of these changes (Figure 2). We identified that both the amplitude of LAI during the rising phase (April to July) and the falling phase (July to October) showed an increasing trend for all vegetation types (Figure S1). During the early stage of canopy development, vegetation (VLAI) developed rapidly. In April and May, VLAI showed an increase, accounting for 71.85% and 75.36% of the regions, respectively. Among these increases, VLAI exhibited a significant increase in May, accounting for 27.54% of the regions. As vegetation grew, the speed of canopy development slowed down. In June, July, and August, the proportion of areas with significant increases in VLAI was 63.99%, 61.07%, and 25.09%, respectively. Notably, the VLAI primarily exhibited a decreasing trend in August. VLAI exhibited a significant decrease in August, accounting for 25.46% of the regions. This suggests that the LAI in July continues to rise, thereby narrowing the gap with the LAI in August and indicating an earlier maturation of the canopy.

There were spatiotemporal differences in LAI and VLAI trends among different vegetation types. From April to August, the earliest significant increase in annual LAI for forests occurred in April, followed by grasslands in May and farmlands in July, accounting for 46.16%, 47.36%, and 71.54% of the areas, respectively. Additionally, from April to August, significant increases in VLAI for grasslands and forests first appeared in May, earlier than for farmlands (June and July), and all were later than April (the initial canopy stage). Specifically, in May, 33.00% of grassland areas showed a significant increase in VLAI, with an average annual increase of 5.06 × 10^−3^ m^2^/m^2^/year, mainly distributed in the eastern part of the Hulunbuir Plateau and the northern part of the Inner Mongolia Plateau. For forests, 29.72% of the areas showed a significant increase in VLAI, with an average annual increase of 27.87 × 10^−3^ m^2^/m^2^/year, mainly concentrated in the high-latitude regions of the Greater and Lesser Khingan Mountains (Figure S4 and Figure 1). In June, 54.52% of farmland areas showed a significant increase in VLAI, with an average annual increase of 18.53 × 10^−3^ m^2^/m^2^/year (Figure S4). In July, 51.56% of farmland areas exhibited a significant increase in VLAI, with an average annual increase of 47.55 × 10^−3^ m^2^/m^2^/year (Figure S4). Compared to forests and grasslands, changes in LAI and VLAI were more significant in farmlands during June and July. In August, the annual increase in LAI was smaller for all vegetation types. Specifically, 15.74% of forest areas experienced a significant decrease in LAI, with an average annual decrease of −25.67 × 10^−3^ m^2^/m^2^/year, mainly distributed in the mountainous regions of the western Greater Khingan Mountains and the southern Sanjiang Plain (Figures S3 and S5). Simultaneously, VLAI also showed a decreasing trend in August, especially for forests and farmlands, with average annual decreases of −57.72 × 10^−3^ and −51.32 × 10^−3^ m^2^/m^2^/year, respectively (Figures S4 and S6). Specifically, 40.06% and 31.42% of farmland and forest areas, respectively, showed a significant decrease in VLAI in August, mainly distributed in high-latitude and high-altitude regions.

During the canopy senescence phase, the increase in the LAI of vegetation in September was much greater than in October. The increases in September for grasslands, forests, and farmlands were 5.29, 3.14, and 4.51 times higher than those in October, respectively (Figure S2). Additionally, the increasing trend area of VLAI for vegetation was much larger in September (65.72%) than in October (17.07%). Among them, forests had the largest increasing VLAI area in September (90.41%) (Figure S6), with a significant increase in area accounting for 28.32%, and an average annual increase of 40.33 × 10^−3^ m^2^/m^2^/year (Figure S4), indicating a significant slowdown in vegetation senescence. Farmlands and grasslands showed no significant increase in VLAI in September, with an area accounting for less than 54% (Figure S6), and a significant increase in area of less than 11%. In October, the VLAI of the three vegetation types showed a decreasing trend, with an area accounting for more than 71%, indicating accelerated vegetation senescence, especially for farmlands and grasslands (87.43% and 93.09%) (Figure S6). Among them, farmlands showed a significant decrease in VLAI area in October, accounting for 49.05%, with an average annual decrease of −17.18 × 10^−3^ m^2^/m^2^/year (Figure S4). Grassland VLAI showed a significant decrease in area, accounting for 40.77%, with an average annual decrease of −8.17 × 10^−3^ m^2^/m^2^/year (Figure S4), mainly distributed in high-latitude areas. However, forests showed a relatively small significant decrease in area in October (18.60%), mainly distributed in the low-latitude area of the Changbai Mountains.

Some scholars have used the difference in the NDVI values of two continuous months as an indicator [36,37]. They analyzed the development and senescence of vegetation canopies in temperate regions of China and the Qinghai–Tibet Plateau. During the early stages of canopy development, the vegetation canopies’ development in all three regions exhibited an accelerating trend. Studies based on the Qinghai–Tibet Plateau and temperate regions of China have addressed the regional differences in vegetation canopy development changes. However, they have not conducted further comparative analysis within vegetation types. Our research identified that the canopy developmental rate of croplands reached its peak during the mid-canopy canopy developmental stage (June), rather than the early stage (May). Additionally, forests and croplands exhibited a more pronounced trend of early canopy maturity in August, which was not observed in grasslands. Lastly, during the canopy senescence stage, forests exhibited the most delayed senescence trend compared to other vegetation types.

### 2.2. Partial Correlation Analysis Between VLAI (or LAI) and Climatic Factors

**Figure 3:**
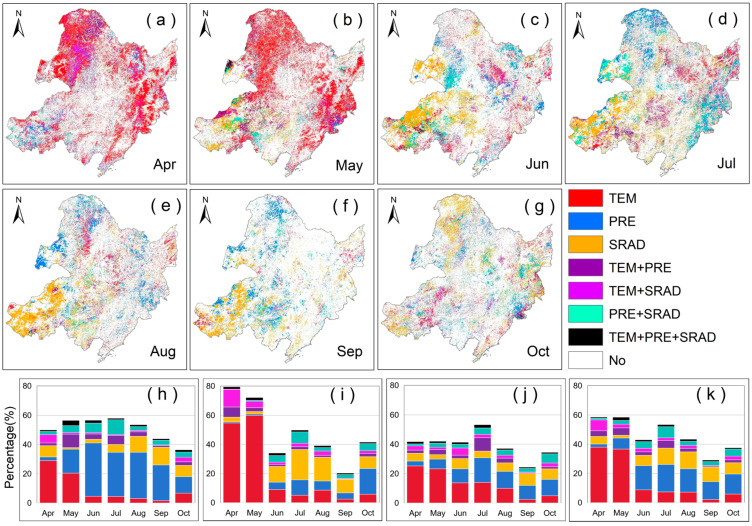
Spatial patterns of the partial correlation between the LAI and preseason climatic factors in Northeast China (with monthly designations: (**a**) = April, (**b**) = May, (**c**) = June, (**d**) = July, (**e**) = August, (**f**) = September, (**g**) = October and statistical chart of percentage of regions among different factors for grasslands (**h**), forests (**i**), farmlands (**j**), and three vegetation types (**k**). White bars show the fraction of insignificant, colored bars show the fraction of significant partial correlations at *p* < 0.05. Among them, “No” indicates that the area is not significantly affected by preseason factors; “TEM”, “PRE”, and “SRAD” indicate that the area is significantly affected by a single preseason factor; “TEM + PRE”, “TEM + SRAD”, and “PRE + SRAD” indicate that the area is significantly affected by two preseason factors; “TEM + PRE + SRAD” indicates that the area is significantly affected by all three preseason factors.

**Figure 4:**
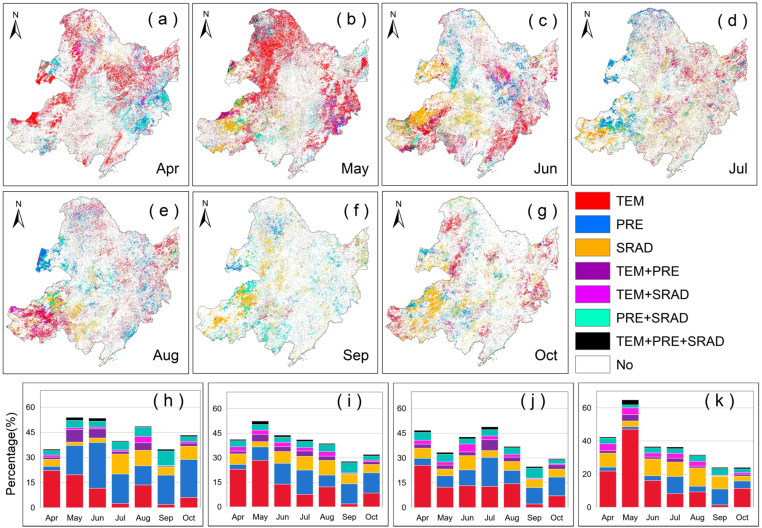
Spatial patterns of the partial correlation between the VLAI and preseason climatic factors in Northeast China(with monthly designations: (**a**) = April, (**b**) = May, (**c**) = June, (**d**) = July, (**e**) = August, (**f**) = September, (**g**) = October and statistical chart of percentage of regions among different factors for grasslands (**h**), forests (**i**), farmlands (**j**), and three vegetation types (**k**). White bars show the fraction of insignificant, colored bars show the fraction of significant partial correlations at *p* < 0.05. Among them, “No” indicates that the area is not significantly affected by preseason factors; “TEM”, “PRE”, and “SRAD” indicate that the area is significantly affected by a single preseason factor; “TEM + PRE”, “TEM + SRAD”, and “PRE + SRAD” indicate that the area is significantly affected by two preseason factors; “TEM + PRE + SRAD” indicates that the area is significantly affected by all three preseason factors.

We conducted a partial correlation analysis between VLAI (or LAI) and preseason climatic factors to explore the response characteristics of vegetation canopy changes to climate change. The spatial patterns where LAI (or VLAI) is significantly affected by preseason climatic factors, along with their statistics, are shown in Figure 3 and Figure 4. In Northeast China, the LAI and VLAI of vegetation in more areas are significantly affected by preseason TEM (22.08% and 19.08%), followed by preseason PRE (20.37% and 17.49%), and preseason SRAD has the least impact (15.41% and 13.32%). Among them, the significant influence of preseason TEM on the development stage of the vegetation canopy (37.20% and 28.92%) is greater than that on the canopy maturity stage (14.13% and 16.19%), and the canopy senescence stage (7.34% and 7.21%). However, the same preseason climatic factor has different effects on different vegetation types.

#### 2.2.1. Partial Correlation Analysis Between VLAI (or LAI) and Precipitation

**Figure 5:**
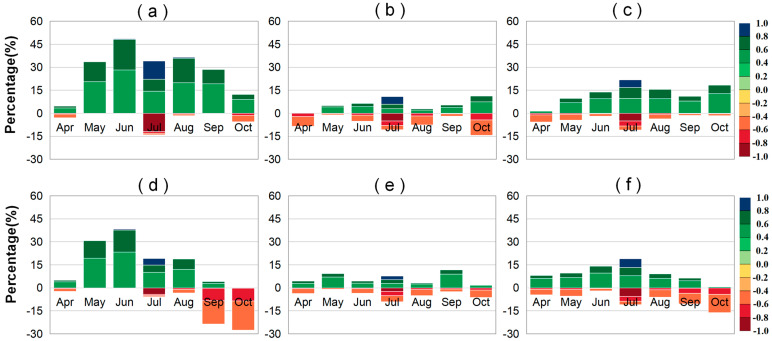
Stacked percentage chart of regions where the different significant partial correlation (*p* < 0.05) ranges between the LAI (or VLAI) and preseason PRE factors for grasslands (**a**,**d**), forests (**b**,**e**), and farmlands (**c**,**f**) in Northeast China. The upward and downward bars represent percentages of positive and negative correlations, respectively.

The statistical chart showing the percentages of regions where the monthly LAI and VLAI of grasslands, forests, and farmlands are significantly affected by preseason PRE in Northeast China is presented in Figure 5. Among the three vegetation types, preseason PRE has the greatest impact on grasslands, followed by farmlands, and the least impact on forests (Figures S10 and S11). The average monthly areas where LAI is significantly affected account for 32.11%, 17.42%, and 13.14%, respectively, while the average monthly areas where VLAI is significantly affected account for 25.83%, 17.54%, and 10.57%, respectively.

For grasslands, there was a significant positive correlation between preseason PRE and VLAI in a large area from April to August, accounting for more than 68%, mainly distributed in the western parts of the Inner Mongolia Plateau and Hulunbuir Plateau (Figure S11). Among them, the significant impact of preseason PRE on grassland VLAI in May and June (31.47% and 38.75%) was greater than in July and August (24.96% and 22.49%). For forests and farmlands, preseason PRE mainly showed a significant positive correlation with VLAI from April to June, accounting for more than 54% of the significantly correlated areas. Among them, for farmlands, the area where preseason PRE showed a significant positive correlation with VLAI in June is the largest (14.20%), mainly distributed in the southwestern agricultural planting area, while the month with the largest area where forest VLAI was significantly positively affected by preseason PRE was earlier than that of farmlands (May: 9.38%), mainly distributed in the northern mountainous area of the Greater Khingan Range (Figure S11).

From September to October, the areas where grassland, farmland, and forest VLAI were significantly negatively affected by preseason PRE gradually increased, especially in October (27.62%, 16.16%, and 6.22%), mainly distributed in the western part of the study area and higher altitude mountainous areas (Figure S11). Among them, in September, unlike grasslands and farmlands, the area where forest VLAI showed a positive correlation with preseason PRE (11.81%) was much larger than the area where it showed a negative correlation with preseason PRE (2.41%) (Figure 5). The significantly positively correlated areas are mainly distributed in the western mountainous area of the Greater Khingan Range (Figure S11).

Overall, the increase in preseason PRE promotes vegetation growth during the early stages of canopy development and accelerates vegetation senescence during the late stages of canopy senescence, especially in the drier western regions. However, due to different vegetation types, the impact of preseason PRE on VLAI varies from July to September. Among them, from July to August, preseason PRE mainly showed a positive correlation with farmland and grassland VLAI, which was earlier than the time when it mainly showed a positive correlation with forest VLAI (September).

#### 2.2.2. Partial Correlation Analysis Between VLAI (or LAI) and Air Temperature

**Figure 6:**
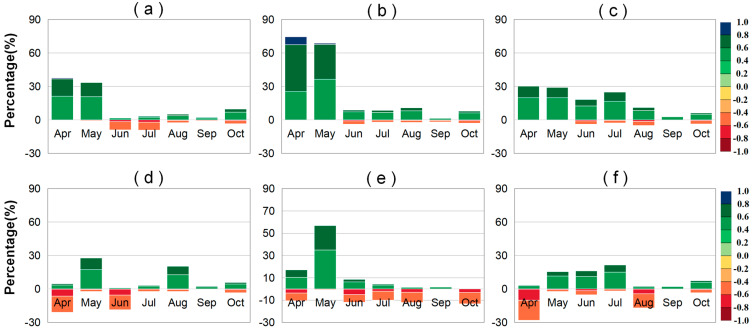
Stacked percentage chart of regions where the different significant partial correlation (*p* < 0.05) ranges between the LAI (or VLAI) and preseason TEM factors for grasslands (**a**,**d**), forests (**b**,**e**), and farmlands (**c**,**f**) in Northeast China. The upward and downward bars represent percentages of positive and negative correlations, respectively.

The statistical chart showing the percentages of regions where monthly LAI and VLAI of grasslands, forests, and farmlands are significantly affected by preseason TEM in Northeast China is presented in Figure 6. Among the three vegetation types, preseason TEM has the greatest impact on forest growth, followed by farmlands, and the least impact on grasslands (Figures S10 and S11). Specifically, the average monthly areas significantly affected by LAI account for 27.72%, 20.05%, and 17.14%, respectively, and the average monthly areas significantly affected by VLAI account for 21.64%, 18.32%, and 16.66%, respectively.

My study finds that the months when preseason TEM significantly affects VLAI are similar to those when VLAI changes significantly (Figures S2 and S8). From April to July, the months when more than 15% of the three vegetation types’ VLAI was significantly affected by preseason TEM corresponded one-to-one with the months when the VLAI significantly increased by more than 15%. Specifically, in May, the forest VLAI had the largest significantly positively correlated area with preseason TEM (57.35%) (Figure S8); from May to July, the farmland VLAI had significantly positively correlated areas with preseason TEM accounting for 15.49%, 16.34%, and 21.76%, mainly distributed in high-latitude agricultural planting areas (Figure S11); in May, 28.08% of the grassland VLAI was significantly positively correlated with preseason TEM, mainly distributed in the Inner Mongolia Plateau (Figure S11). Additionally, in August, the VLAI of farmlands and forests decreased significantly. At the same time, the areas where farmland and forest VLAI were negatively correlated with preseason TEM were the largest in August (72.40% and 64.57%) (Figure S8), with significantly negatively correlated areas accounting for 17.05% and 12.07%, respectively. Finally, from September to October, different vegetation types were less significantly affected by preseason TEM, with regional proportions of less than 4% and 15%, respectively. Specifically, the positively correlated area of forest VLAI with preseason TEM in September (71.00%) was much larger than that of grasslands (48.47%) and farmlands (53.88%) (Figure S8), and the negatively correlated area of forest VLAI with preseason TEM in October (79.84%) was also much larger than that of grasslands (40.00%) and farmlands (39.54%) (Figure S8). The VLAI of forests showed a significant increasing trend in September and a significant decreasing trend in October (Figure S2), which are similar to the effects of preseason TEM on forest VLAI.

It is worth noting that during the canopy growth period from April to July, some vegetation types were negatively affected by preseason TEM. In April, preseason TEM was mainly significantly negatively correlated with grassland and farmland VLAI (20.91% and 28.09%), and these areas accounted for 81.32% and 88.58% of the significantly affected areas, mainly distributed in high-latitude farmlands, the western part of grasslands, and the agropastoral ecotone (Figure S11). Except for April, as vegetation grew, the areas where vegetation VLAI was negatively affected by preseason TEM began to increase. The negatively correlated areas of forest VLAI with preseason TEM gradually increased from June to July (35.83% and 58.68%) (Figure S8), and in July, the significantly negatively correlated areas of VLAI with preseason TEM (10.15%) accounted for 69.90% of the significantly affected areas. The proportion of negatively correlated areas of grassland VLAI with preseason TEM exceeded 61% (Figure S8) from June to July, and in June, the significantly negatively correlated areas of VLAI with preseason TEM (18.67%) accounted for 93.21% of the significantly affected areas.

#### 2.2.3. Partial Correlation Analysis Between VLAI (or LAI) and Solar Radiation

**Figure 7:**
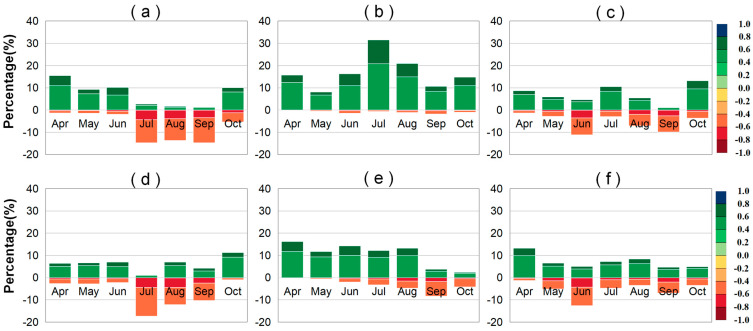
Stacked percentage chart of regions where the different significant partial correlation (*p* < 0.05) ranges between the LAI (or VLAI) and preseason SRAD factors for grasslands (**a**,**d**), forests (**b**,**e**), and farmlands (**c**,**f**) in Northeast China. The upward and downward bars represent percentages of positive and negative correlations, respectively.

The statistical chart showing the percentages of regions where monthly LAI and VLAI of grasslands, forests, and farmlands are significantly affected by preseason SRAD in Northeast China is presented in Figure 7. Among the three vegetation types, preseason SRAD has the greatest impact on the growth of forests, followed by grasslands, and the least impact on farmlands (Figures S10 and S11). Specifically, the average monthly areas where LAI is significantly affected account for 17.93%, 14.88%, and 12.68%, respectively, and the average monthly areas where VLAI is significantly affected account for 13.97%, 13.20%, and 12.57%, respectively.

Vegetation VLAI and preseason SRAD mainly show a positive correlation. During the seven-month vegetation growth phase, the months where the VLAI of grasslands, farmlands, and forests showed a significant positive correlation with preseason SRAD more than a significant negative correlation contained 4, 5, and 5 months, respectively. During these periods, the average monthly area with a significant positive correlation was largest for forests (13.61%), followed by farmlands (8.11%), and smallest for grasslands (7.86%). For forests, in September and October, VLAI and preseason SRAD mainly showed a negative correlation (73.29% and 62.59%) (Figure S9), with the significantly negatively correlated area gradually decreasing from 8.39% to 4.19%, mainly distributed in the Greater Khingan region (Figure S11). For grasslands, from July to September, VLAI was significantly affected by preseason SRAD (18.57%, 19.21%, and 14.47%), mainly showing a significant negative correlation, accounting for more than 63% of the significantly correlated areas. An increase in preseason SRAD can inhibit grassland growth. This inhibitory effect gradually decreased in the later stages of growth, with the proportion of significantly negatively correlated areas decreasing from 17.37%, 12.16%, and 10.25% from July to September, mainly located in the western grasslands’ growth area (Figure S11). For farmlands, in June and September, the areas where VLAI and preseason SRAD showed a negative correlation were larger (59.86% and 55.59%) (Figure S9), with significantly negatively correlated areas (12.63% and 6.97%) accounting for 71.62% and 59.94% of the significantly correlated areas, respectively. This significant negative correlation is mainly located in the agricultural planting areas of Heilongjiang Province in June and Liaoning Province in September (Figure S11). Additionally, June is the month where VLAI was most significantly affected by preseason SRAD (17.64%).

### 2.3. Lagged Effect of Climate Factors on LAI and VLAI

**Figure 8:**
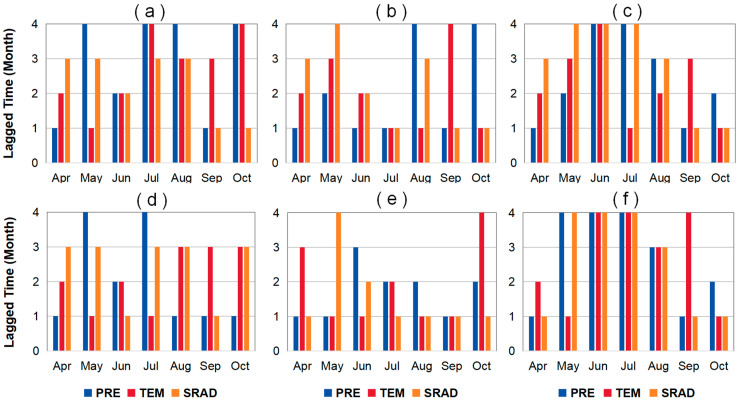
The length of month with the highest partial correlation coefficients between monthly LAI (or VLAI) and climate variables (PRE, TEM, and SRAD) for grasslands (**a**,**d**), forests (**b**,**e**), and farmlands (**c**,**f**).

Based on the monthly averages of LAI, VLAI, and three preseason climate factors for grassland, forest, and farmland in Northeast China, we conducted partial correlation analysis to identify the preseason month when LAI and VLAI showed the strongest partial correlation with climate factors (Figure S14). Based on this, we analyzed the differences in the lagged effect of climatic factors on LAI and VLAI for different vegetation types by calculating the percentage of the number of preseason months (including 1, 2, 3, and 4 months) out of the total preseason months at different time periods. A larger preseason month number indicates a greater lagged effect of preseason climatic factors. The statistics of the length of the month with the highest partial correlation coefficients between LAI (or VLAI) and climatic factors for the three vegetation types are shown in Figure 8. For grasslands, the lagged effect of climatic factors on LAI and VLAI mainly occurs within 1 and 3 months (30.95% and 33.33%), and this variation is particularly evident during the canopy senescence period (September–October) (38.89% and 44.44%). For forests, the lagged effect primarily occurs within 1 month (52.38%) and is inversely proportional to the number of preseason months, accounting for 21.43%, 11.90%, and 14.29% within 2, 3, and 4 months, respectively. This variation is similar during both the canopy development period (April–August) and the senescence period. Specifically, during the canopy development and senescence periods, the proportion of one preseason month is 45.83% and 61.11%, respectively. For farmlands, the lagged effect mainly occurs within 1 and 4 months (30.95% and 35.71%), but this variation differs significantly between the canopy development and senescence periods. During the canopy development period, the lagged effect primarily occurs within 4 months (58.33%), while during the canopy senescence period, it mainly occurs within 1 month (44.44%).

**Figure 9:**
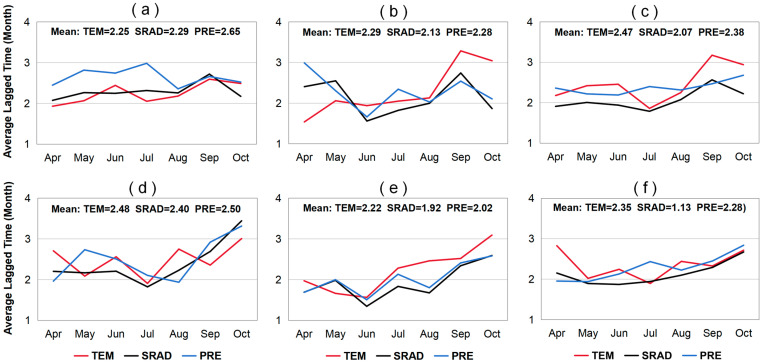
Average lagged time with the highest simple correlation coefficients between monthly LAI (or VLAI) and climate variables (PRE, TEM, and SRAD) for grasslands (**a**,**d**), forests (**b**,**e**), and farmlands (**c**,**f**).

There are spatiotemporal differences in the preseason months corresponding to LAI (or VLAI) and climate factors for different vegetation types (Figures S12 and S13). We calculated the average lagged time (average preseason month value) for each vegetation type using area as the weight, as shown in Figure 9. A longer average lagged time indicates a greater lagged effect of climatic factors on the vegetation. Among the three vegetation types, climatic factors have the strongest lagged effect on the LAI and VLAI of grasslands (2.40 and 2.46 months), followed by farmlands (2.31 and 2.26 months), and the effect on forests is the smallest (2.24 and 2.05 months). Additionally, the average lagged effect of climatic factors on the VLAI for grasslands, farmlands, and forests during the later growth period (August–October) is greater (2.74, 2.45, and 2.39 months) than during the early growth period (April–July) (2.25, 2.11, and 1.81 months). Finally, different climatic factors have a varying lagged effect on VLAI for the same vegetation type. For grasslands, PRE has the greatest average lagged effect on VLAI (2.50 months), followed by TEM (2.48 months), and SRAD has the smallest effect (2.40 months). For farmlands and forests, TEM has the greatest average lagged effect on VLAI (2.35 and 2.22 months), followed by PRE (2.28 and 2.02 months), and SRAD has the smallest effect (2.13 and 1.92 months).

## 3. Discussion

### 3.1. Vegetation Canopy Development Changes at Finer Temporal Scale

Recent studies have indicated a significant weakening trend in the connection between summer greening and spring greening in the Northern Hemisphere [30], and our research supports this viewpoint. From April to August, the rate of increase in the VLAI is faster during the early stage than in the later stage in Northeast China. By August, when the canopy reaches maturity, there is a significant decrease in VLAI for both forests and farmlands, indicating a notable slowdown in vegetation development. Specifically, both LAI and VLAI show a significant decreasing trend in forests in August, which is consistent with previous studies [36,42]. This could be explained by the high demand for carbohydrates during the early stages of canopy development. Plants tend to invest carbon into leaves for photosynthesis, thereby increasing carbohydrate production. However, warmer and more extreme hot weather is not conducive to vegetation growth during the peak season, shifting the peak of plant growth to an earlier period and resulting in early maturation of the vegetation [30]. This change might be related to the carbon allocation strategy of vegetation at different growth stages and the negative feedback mechanism between photosynthesis and canopy development caused by carbon surplus. Studies have shown that as the TEM rises in the later growth stages and the vegetation gradually matures in the middle and late growing season, the carbon consumption of its autotrophic respiration will increase, even exceeding photosynthesis [43]. At this time, it is uneconomical to continue to allocate a large amount of carbon to the leaves [44,45]. Besides the change in the carbon allocation mechanism, which reduces the proportion of carbon allocated to leaves, when the photosynthetic output exceeds the carbon required for vegetation growth, the excess carbon will accumulate in the leaves. This accumulation can also reduce the rate of photosynthesis, leading to accelerated leaf abscission [36,46,47].

During the canopy senescence stage, there is substantial evidence indicating a delayed trend at the end of the vegetation growing season with global warming [26,48]. Similar to the initial stage of canopy development, our study identified that the slower canopy senescence in September is related to the delay in autumn phenology due to warming, especially in forests, where 71.00% of the areas show a positive correlation with preseason TEM. However, in October, with increasing preseason TEM, the VLAI in forests shows a decreasing trend (79.84%). Studies have suggested that the increase in leaf fall during the last month of the growing season can be explained by the increase in canopy greenness resulting from reduced canopy senescence earlier. It is worth noting that although the VLAI changes in forests and farmlands are similar from August to October, the delaying effect of canopy senescence is more pronounced in forests. This is partly because forests experience rapid growth in spring and slow decline in autumn, whereas farmlands experience slow growth and rapid decline. Additionally, forests exhibit slow color changes, mixed colors, and difficulty in detection during the canopy senescence stage, while changes in farmland vegetation maturation and harvesting are very evident. Furthermore, in September, there are latitudinal differences in the delaying effect of increasing preseason TEM on vegetation senescence. The increasing trend of LAI is stronger in low-latitude areas than in high-latitude areas. This could be because in higher altitude and lower TEM regions, the EOS of vegetation advances to avoid the dangers of frost [49].

The phenological stages of vegetation, as predicted by remote sensing, show a strong linear correlation with survey-derived datasets. Studies have utilized field observations from phenological networks in the United States and Europe to validate satellite-derived land surface phenology [20]. They identified that the onset of vegetation green-up, detected by over 70% of remote sensing data sampling points, aligns with multi-year anomalies and long-term trend directions observed in situ. In cold biomes, long-term records indicate that climate warming has led to a phenomenon of earlier spring growth and/or later autumn senescence [50]. Among these, temperate forests exhibit the most pronounced trends: earlier spring phenology (from budburst to full leaf expansion) and delayed autumn phenology (from senescence to complete leaf fall) [12]. However, crop phenology is more complex, being regulated not only by natural factors (e.g., climate) but also by intensive management practices (e.g., decisions on crop varieties and sowing dates) [51]. Research has indicated that in North America, the planting date for crops has advanced, and the harvest date has been delayed [52]. Specifically, the increase in minimum temperature leads to advancement in planting time or delay in harvest time. Higher maximum temperatures, on the other hand, result in advancement in planting time or advancement in harvest time. Lastly, for regions experiencing water stress, the earlier onset of the thermal growing season enhances vegetation growth in cold and humid areas, but not in arid regions [53]. These findings align with similar patterns observed in our study.

### 3.2. Impact of Preseason Climate Factors on VLAI Changes

Research has shown that in the past few decades, the SOS in China has advanced [9,54], and our study observed a similar trend with accelerated canopy development across different vegetation types. Furthermore, we identified that as vegetation grows, the negative impact of preseason TEM on different vegetation types begins to increase. This overall trend in VLAI variation is similar to the effect of increasing preseason TEM on VLAI. It is worth noting that the negative impact of preseason TEM on arid and water-scarce grassland areas in the west occurs earlier than in forests and farmlands. This might be due to the increased transpiration caused by earlier vegetation greening as a result of spring warming, leading to a significant soil moisture deficit in summer [55,56,57]. In such cases, plants tend to invest more carbon into their root systems during the late greening stage to acquire water and nutrients, thus increasing the carbon cost per unit of leaf area, which is unfavorable for canopy growth in the region [58,59,60,61]. Therefore, we believe that the accelerated development of the spring vegetation canopy consumes additional resources needed to maintain subsequent growth [30], intensifying water stress on the vegetation, resulting in a decrease in net photosynthetic rate and a shift in carbon allocation from leaves to stems [62], and thereby negatively affecting grassland VLAI.

Moreover, PRE not only directly affects vegetation phenology but also indirectly influences it by regulating radiation and heat demand, especially in arid and semi-arid grassland regions [26]. Our study revealed that preseason PRE has a significant impact on grasslands (25.54%), which is greater than that on forests (12.57%) and farmlands (8.19%), particularly in May and June. As the summer monsoon brings abundant rain in July and August, the pressure between reduced water supply and increased water demand in grasslands is alleviated, and the negative impact of preseason TEM on vegetation gradually diminishes. This suggests that grassland is most sensitive to combined hydrothermal conditions, and as regional drought severity decreases, the influence of preseason TEM on vegetation phenology increases. These findings align with the view that vegetation in most temperate regions of the Northern Hemisphere (30° N–50° N), especially in arid areas, is primarily controlled by water supply [49,63].

Among the three climate factors, preseason SRAD has a relatively small impact on vegetation growth, with a closer association observed in forest growth, consistent with previous studies [23]. During the canopy development stage, preseason SRAD mainly promotes vegetation growth, as adequate SRAD is necessary to initiate growth. However, as spring transitions into summer, the areas where increased SRAD promotes vegetation growth decrease, while regions where it inhibits growth increase, particularly in the western arid zones and agricultural planting areas. This could be due to the damaging effects of excessive UV-B radiation from strong SRAD on plant *DNA(Deoxyribonucleic Acid)*, *proteins*, and *membranes*, negatively impacting photosynthesis and growth [64].

### 3.3. Uncertainties and Future Directions

In agriculture, forestry, and pastoral industries, our research on the growth and change of the canopy in different vegetation types provides crucial data for the design and experimentation of plant growth and development models. Furthermore, the study on the response characteristics of canopy growth of different vegetation types to climate change holds significant importance for vegetation planting decisions, irrigation strategies, pest and disease control, land-use planning, and ecological restoration. To better understand canopy changes in vegetation, several methods can be employed in the future to further refine experiments and enhance research accuracy. Firstly, the information at a single pixel in remote sensing imagery is often not independent and can be influenced by neighboring areas [65]. Pixel-by-pixel methods primarily focus on local information at each pixel, neglecting the global relationships between pixels. Therefore, texture information is frequently utilized in vegetation analysis [65,66].

Additionally, partial correlation analysis aligns well with linearly correlated data. However, the adaptation mechanisms of vegetation phenology to environmental changes are complex and nonlinear. With advancements in statistics and computer technology, advanced data processing and analysis tools such as machine learning algorithms and deep learning models can better handle these nonlinear relationships. These tools find applications in constructing knowledge graphs [67], optimizing computing resources [68], image classification [69], and object detection [70]. Consequently, to explore and comprehend the mechanisms of surface phenological changes, it is essential to incorporate these advanced methods into vegetation growth models.

Lastly, differences exist in the canopy change information obtained from vegetation monitoring networks at different “sky–air–ground” scales. These differences can impact the results of surface phenological change mechanisms [21]. It is noteworthy that low-cost phenological research, encompassing observational records and experimental manipulations, is crucial for our understanding of the mechanisms and impacts of phenological changes in plant populations, species, and communities [71]. Currently, there are studies that combine ground-based phenological records with remote sensing image observations to investigate vegetation phenological changes [72,73,74,75]. Therefore, to enhance the accuracy of extracting canopy growth and change, and to mitigate the influence of scale effects, it is necessary to further analyze the differences in vegetation canopy growth across various spatiotemporal scales in the future.

## 4. Materials and Methods

### 4.1. Study Area

**Figure 10:**
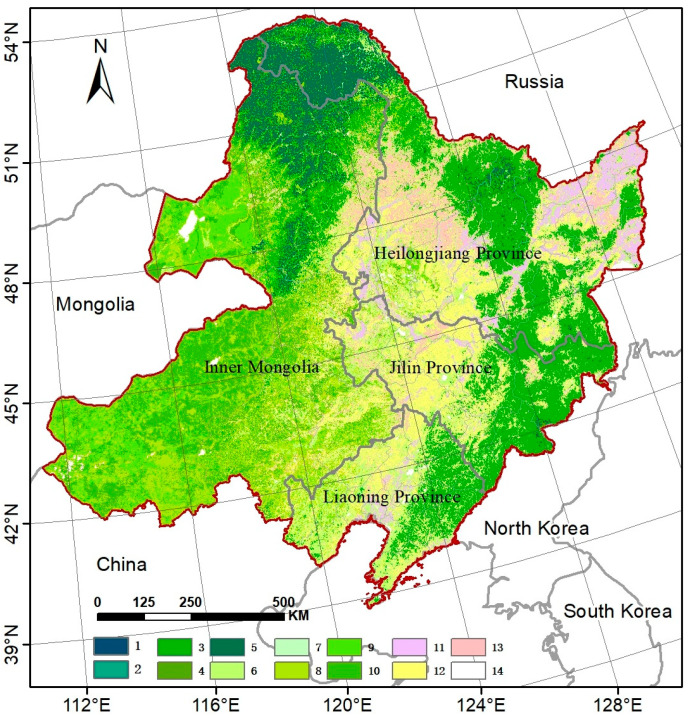
The geographic location and land use pattern across Northeast China. The legend indicates: 1 is coniferous and broad-leaved mixed forest, 2 is evergreen broad-leaved forest, 3 is deciduous broad-leaved forest, 4 is evergreen coniferous forest, 5 is deciduous coniferous forest, 6 is evergreen shrubland, 7 is deciduous shrubland, 8 is low-coverage grassland, 9 is medium-coverage grassland, 10 is high-coverage grassland, 11 is rice paddy, 12 is corn, 13 is soybean, and 14 is others.

Our study area was the Northeast region (110°–136° E and 38°–54° N), including Heilongjiang, Jilin, and Liaoning provinces, and the eastern five leagues of the Inner Mongolia Autonomous Region (including Hulunbuir, Hinggan, Tongliao, Chifeng, and Xilin Gol League) (Figure 10). The region is surrounded by medium and low mountains on the east, west, and north, with a large plain opening southward in the middle. Most of the mountainous areas have an elevation of 1000–1500 m. This region experiences thermal variations from warm temperate, temperate, to cold temperate zones from south to north, and humidity differentiation from wet, semi-wet to semi-arid from east to west, forming a unique vegetation distribution pattern. It is one of the sensitive areas for global change. The main forest types include coniferous forests dominated by *larch*, *Korean pine*, *Scotch pine*, *spruce*, and *fir*, as well as broad-leaved forests dominated by *white birch*, *Betula luminifera*, *Mongolian oak*, and *aspen*. The main crops include *spring corn*, *spring wheat*, *soybeans*, *rice*, and *sorghum*, making it China’s most important commodity grain base [76]. The grasslands mainly consist of dry grasslands, meadow grasslands, saline meadows, and wet meadows.

### 4.2. Datasets

In this study, we used data from GLASS LAI (V6) from 2001 to 2020 to detect changes in vegetation canopy development and senescence [77]. This dataset represents the highest spatial resolution long-term global LAI product currently available, with a spatial resolution of 500 m and a temporal resolution of 8 days, which was later processed into monthly data. Monthly climate data include TEM, PRE, and SRAD. Among them, TEM and PRE data were obtained from the National Earth System Science Data Center of the National Science and Technology Infrastructure of China. These data were generated through Delta spatial downscaling based on the global 0.5° CRU climate dataset and the WorldClim global high-resolution climate dataset, with a spatial resolution of 1 km [78]. SRAD data were obtained from the Terra Climate dataset, which covers global land surface monthly climate and climate water balance from 1958 to 2022, with a spatial resolution of 4638.3 m [79].

Land cover data were obtained from the ESA CCI-LC dataset (ESA Climate Change Initiative and in particular its Land Cover project) spanning from 1992 to 2022, with a spatial resolution of 300 m and comprising 22 categories [80]. Considering that scale effects and land cover changes can lead to variations in remotely sensed vegetation parameter values [81], this study only selected data from 2000 to 2020 where there were no changes in the categories of grasslands, forests, and farmlands. LAI, climate, and land cover data were resampled to 1000 m.

### 4.3. Method

We rely on the research of Piao and calculat *VLAI*_(*t*)_ (Equation (1)) using the difference in LAI between two consecutive months [1]. Positive or negative values of *VLAI*_(*t*)_ indicate the rate of canopy development and senescence, respectively.

*VLAI*_(*t*)_ = *LAI*_*t*_ − *LAI*_*t*−1_ (1)

In this context, *t* represents time (month), where *LAI_t_* and *LAI_t__−_*_1_ denote the LAI for the *t*-th and (*t*−1)-th months of a given year, respectively.

**Figure 11:**
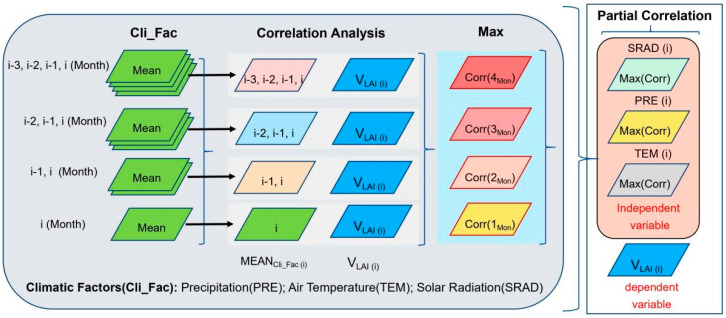
Flowchart of the partial correlation analysis method between LAI_(t)_ (or VLAI_(t)_), and preseason climatic variables. In this context, if TEM(i) represents data from July 2020, TEM(i-1) represents data from June 2020, TEM(i-2) represents data from May 2020, and TEM(i-3) represents data from April 2020.

The vegetation growing season in Northeastern China begins in April and lasts until October. We define the period from April to June as the canopy development stage, July to August as the canopy maturity stage, and September to October as the canopy senescence stage. VLAI values are relatively high during the canopy development and senescence stages. In contrast, VLAI values are relatively low during the canopy maturity stage. Furthermore, we examined the influence of three climatic variables (TEM, PRE, and SRAD) on $LAI_{t}$ and *VLAI*_(*t*)_. As climate may impose a lagged effect on vegetation, we also calculated preseason climatic factors for up to 3 months prior [37]. Specifically, for each pixel, we identified the preseason that exhibited the strongest correlation with monthly *VLAI*_(*t*)_ during the period and conducted a partial correlation analysis using this preseason’s average values. The methodological process is illustrated in Figure 11.

## 5. Conclusions

In this study, we investigated the canopy development (April–June), canopy maturity (July–August), and canopy senescence (September–October) rates in Northeast China and their response characteristics to preseason climatic factors. We identified that the early stages saw acceleration in canopy development, with over 71% of the areas experiencing such accelerates in April and May. As the vegetation grew, the increase in VLAI slowed down, and the canopy maturation phase advanced. By analyzing the partial correlation between VLAI and preseason climatic factors, we identified that changes in VLAI were most significantly affected by preseason air temperature. There was a positive correlation in the early stages of canopy development, gradually transitioning to a negative correlation during canopy maturation and senescence. Notably, the transition timing varied among different vegetation types, with grasslands (June) occurring earlier than forests (July) and farmlands (August). Furthermore, in arid regions, the impact of precipitation on vegetation canopy growth was comparable to that of temperature. Among them, grassland VLAI was more strongly affected by precipitation than that of forests and farmlands, exhibiting the greatest lagged effect of 2.50 months. These findings will enhance our understanding of how vegetation canopies respond to climate change at different growth stages. They provide crucial data support for land-use planning, prevention and control of land degradation, and sustainable agricultural development. Additionally, they are of great significance in strengthening ecosystem monitoring capabilities, improving the accuracy of climate change assessments, and developing viable management practices for climate adaptation and mitigation.

## Data Availability

The original contributions are included in the article/supplementary files. Further queries can be directed to the corresponding author.

## Supplementary Materials

The following supporting information can be downloaded at: https://www.mdpi.com/article/10.3390/plants14010143/s1, Figure S1. Trends in the magnitude of LAI increase and decrease for grasslands (a), forests (b), and farmlands (c) from 2001 to 2020, showing the slope (K value) and correlation coefficient (R2). Both the increase and decrease in LAI show significant trends (*p* < 0.01), Figure S2. Monthly average LAI values and their changes (D_value) for grasslands (a), forests (b), and farmlands (c) from 2001 to 2010 and 2011 to 2020, Figure S3. Trends in LAI for grasslands (a), forests (b), and farmlands (c) from 2001 to 2020, showing the slope (K value). The changes in LAI show significant trends (*p* < 0.01), Figure S4. Monthly trends in VLAI for grasslands (a), forests (b), and farmlands (c) from 2001 to 2020, showing the slope (K value). All trends meet the significance test (*p* < 0.01), Figure S5. Spatial distribution of LAI trends in Northeast China (with monthly designations: a=April, b=May, c=June, d=July, e=August, f=September, g=October). The black squares indicate areas where LAI shows a significant trend (*p* < 0.05), Figure S6. Proportion of areas with different trends in LAI and VLAI for grasslands (a and d), forests (b and e), and farmlands (c and f) in Northeast China, Figure S7. Proportion of areas where LAI and VLAI are affected by preseason precipitation for grasslands (a and d), forests (b and e), and farmlands (c and f) in Northeast China, Figure S8. Proportion of areas where LAI and VLAI are affected by preseason temperature for grasslands (a and d), forests (b and e), and farmlands (c and f) in Northeast China, Figure S9. Proportion of areas where LAI and VLAI are affected by preseason SRAD for grasslands (a and d), forests (b and e), and farmlands (c and f) in Northeast China, Figure S10. Spatial distribution of partial correlations between LAI and preseason climatic factors in Northeast China. The black squares indicate areas where LAI is significantly affected by preseason climate factors (*p* < 0.05), Figure S11. Spatial distribution of partial correlations between VLAI and preseason climatic factors in Northeast China. The black squares indicate areas where VLAI is significantly affected by preseason climate factors (*p* < 0.05), Figure S12. Spatial distribution of preseason months for partial correlation analysis between LAI and climatic factors in Northeast China, Figure S13. Spatial distribution of preseason months for partial correlation analysis between VLAI and climatic factors in Northeast China, Figure S14. Partial correlation statistical graphs between LAI and VLAI of grasslands (a and b), forests (c and d), and farmlands (e and f) and preseason climate factors in Northeast China. Significant correlations (0.01 ≤ *p* < 0.05) with climate factors are marked with “**” and highly significant correlations (*p* < 0.01) are marked with “***.”
