# Supplementary material for: Assessing Vegetation Canopy Growth Variations in Northeast China
Source: Plants (Basel). 2025 Jan 6;14(1):143. doi: 10.3390/plants14010143 (PMC11723273; doi:10.3390/plants14010143)
Supplement: Supplementary file 1 [file plants-14-00143-s001.zip › plants-3359691-supplementary.pdf]

Supplementary Information for

Response of Vegetation Canopy Growth to Climate Change in Northeast China

Lijie Lu[1,2,3], Lingxue Yu[1,*], Xuan Li[1], Li Gao[1], Lun Bao[1], Xinyue Chang[1], Xiaohong Gao[1], Zhongquan Cai[1]

[1] State Key Laboratory of Black Soils Conservation and Utilization, Northeast Institute of Geography and Agroecology, Chinese Academy of Sciences, Changchun, Jilin 130102, China

[2]Faculty of Computing, Harbin Institute of Technology, Harbin 150006, China

[3]National Key Laboratory of Smart Farm Technologies and Systems, Harbin 150006, China

Correspondence: yulingxue@iga.ac.cn

This PDF file contains:

Supplementary Figures S1-S14

Supplementary Figures

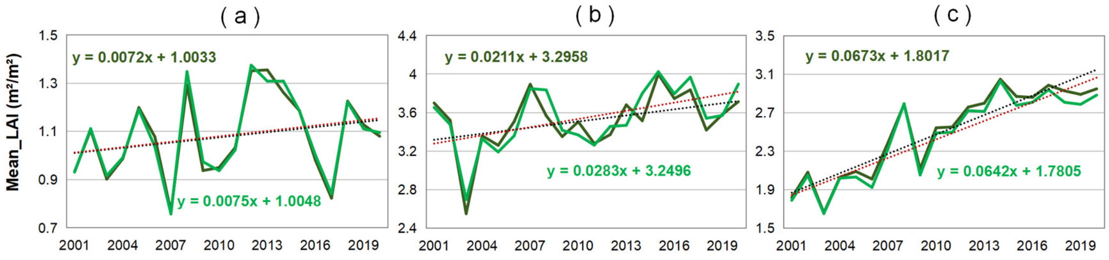

Figure. S1. Trends in the magnitude of LAI increase and decrease for grasslands (a), forests (b), and farmlands (c) from 2001 to 2020, showing the slope (K value) and correlation coefficient (R^2). Both the increase and decrease in LAI show significant trends ($P<0.01$).

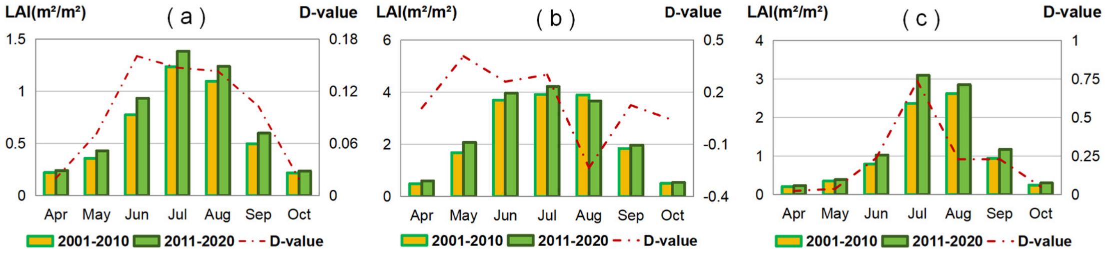

Figure. S2. Monthly average LAI values and their changes(D_value) for grasslands (a), forests (b), and farmlands

(c) from 2001 to 2010 and 2011 to 2020.

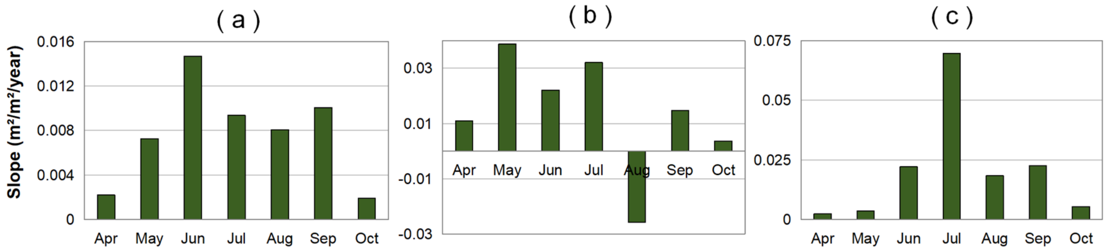

Figure. S3. Trends in LAI for grasslands (a), forests (b), and farmlands (c) from 2001 to 2020, showing the slope (K value). The changes in LAI show significant trends (P<0.01).

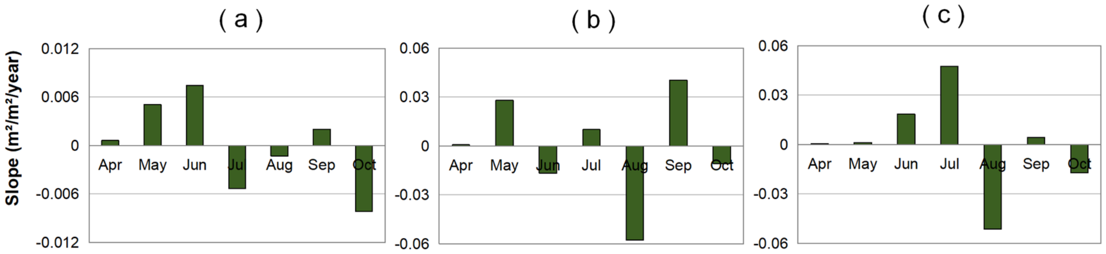

Figure. S4. Monthly trends in VLAI for grasslands (a), forests (b), and farmlands (c) from 2001 to 2020, showing the slope (K value). All trends meet the significance test (P<0.01).

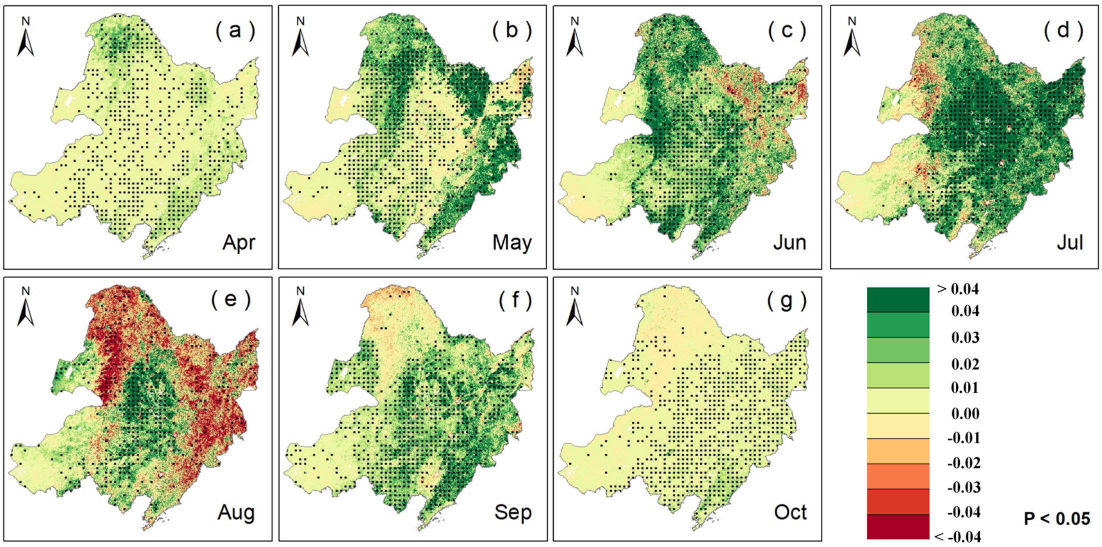

Figure. S5. Spatial distribution of LAI trends in Northeast China (with monthly designations: a=April, b=May, c=June, d=July, e=August, f=September, g=October). The black squares indicate areas where LAI shows a significant trend (P<0.05).

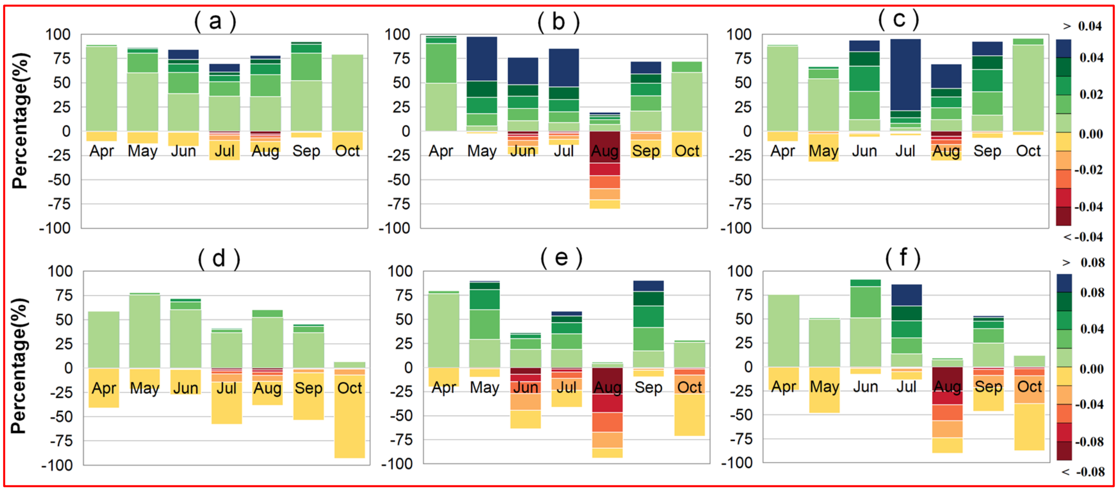

Figure. S6. Proportion of areas with different trends in LAI and VLAI for grasslands (a and d), forests (b and e), and farmlands (c and f) in Northeast China.

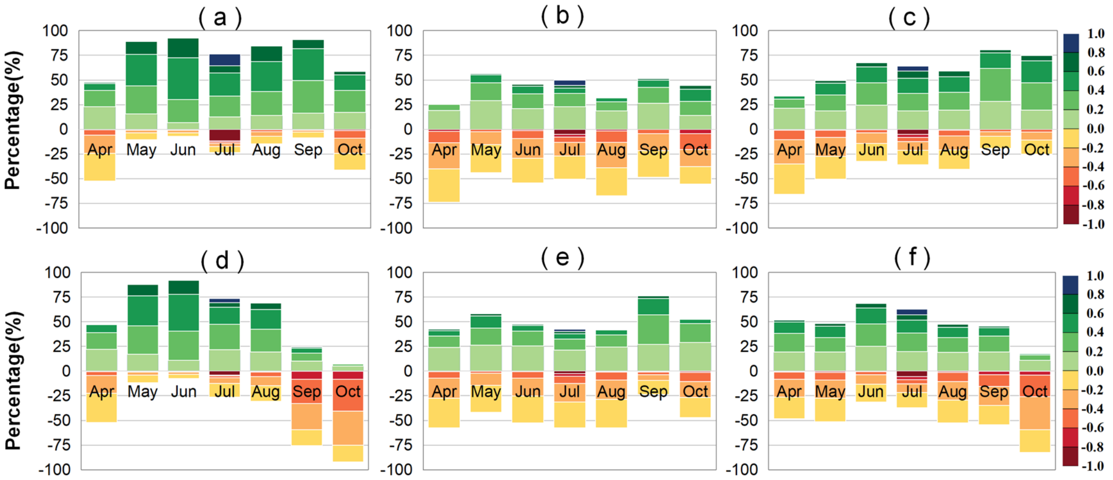

Figure. S7. Proportion of areas where LAI and VLAI are affected by preseason precipitation for grasslands (a and d), forests (b and e), and farmlands (c and f) in Northeast China.

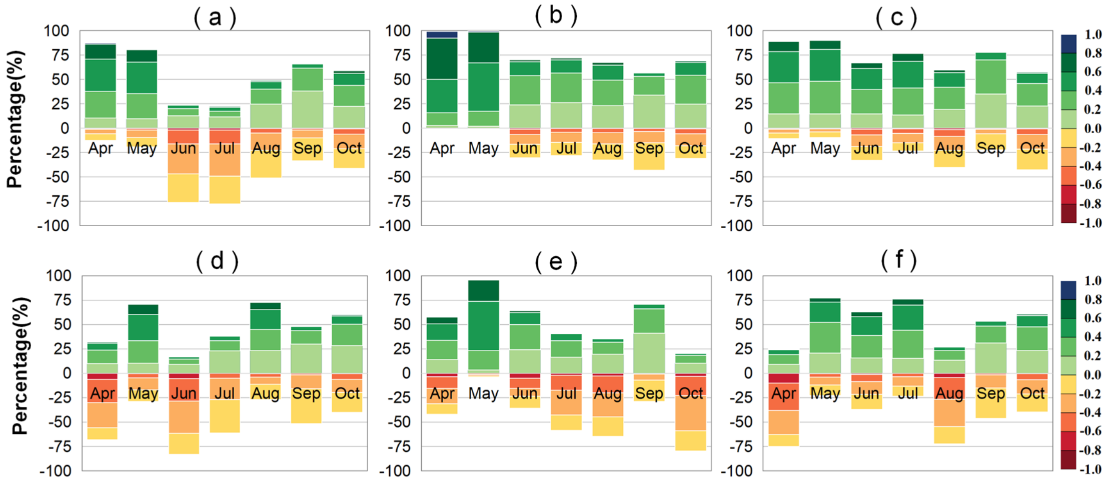

Figure. S8. Proportion of areas where LAI and VLAI are affected by preseason temperature for grasslands (a and d), forests (b and e), and farmlands (c and f) in Northeast China.

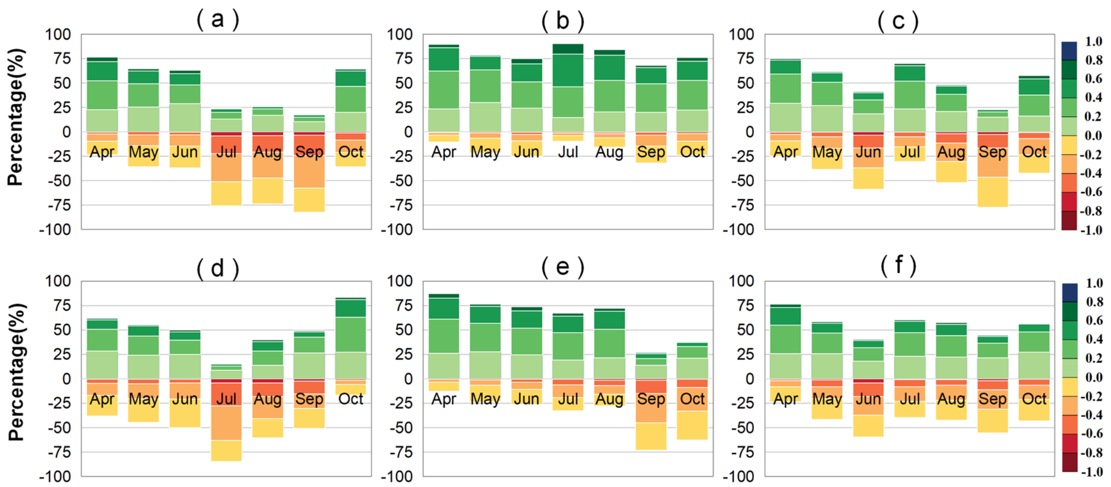

Figure. S9. Proportion of areas where LAI and VLAI are affected by preseason srad for grasslands (a and d), forests (b and e), and farmlands (c and f) in Northeast China.

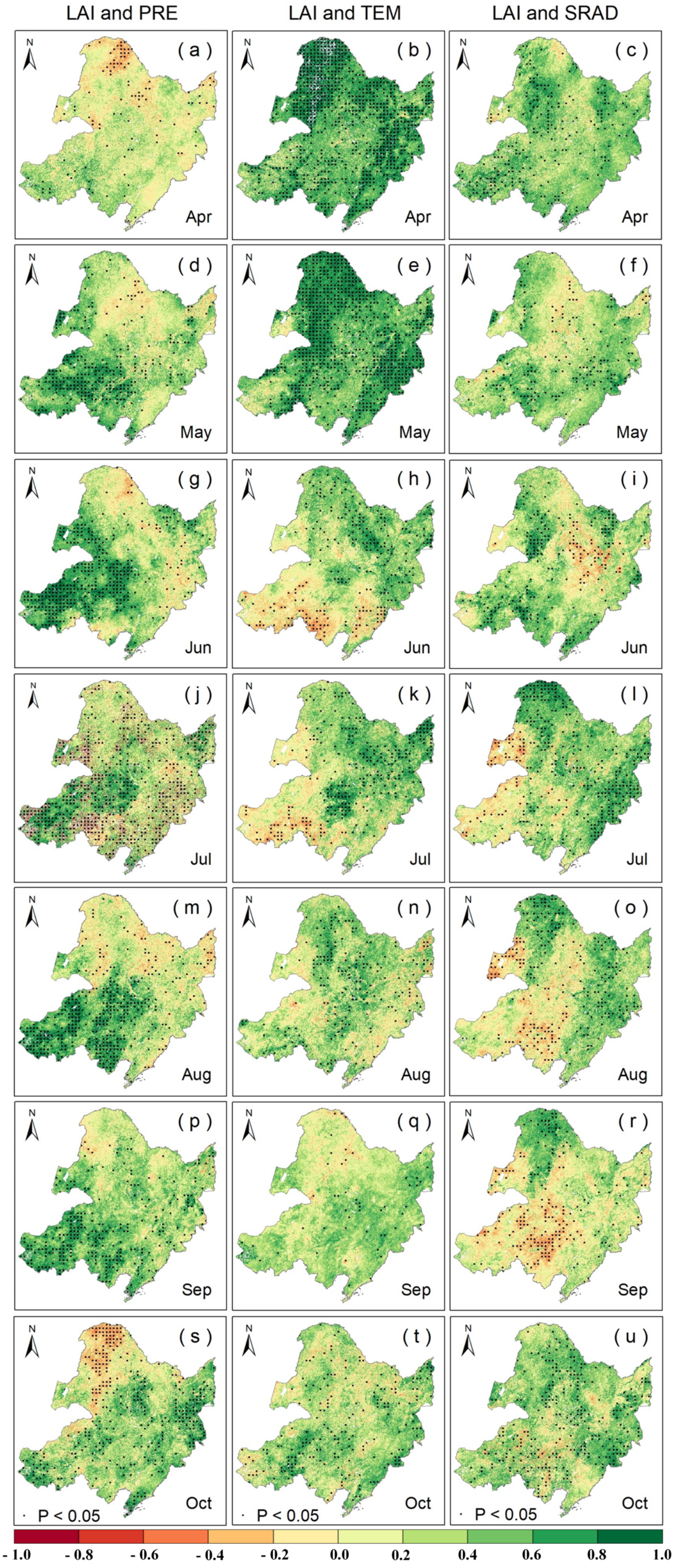

Figure. S10. Spatial distribution of partial correlations between LAI and preseason climatic factors in Northeast China. The black squares indicate areas where LAI is significantly affected by preseason climate factors(P<0.05).

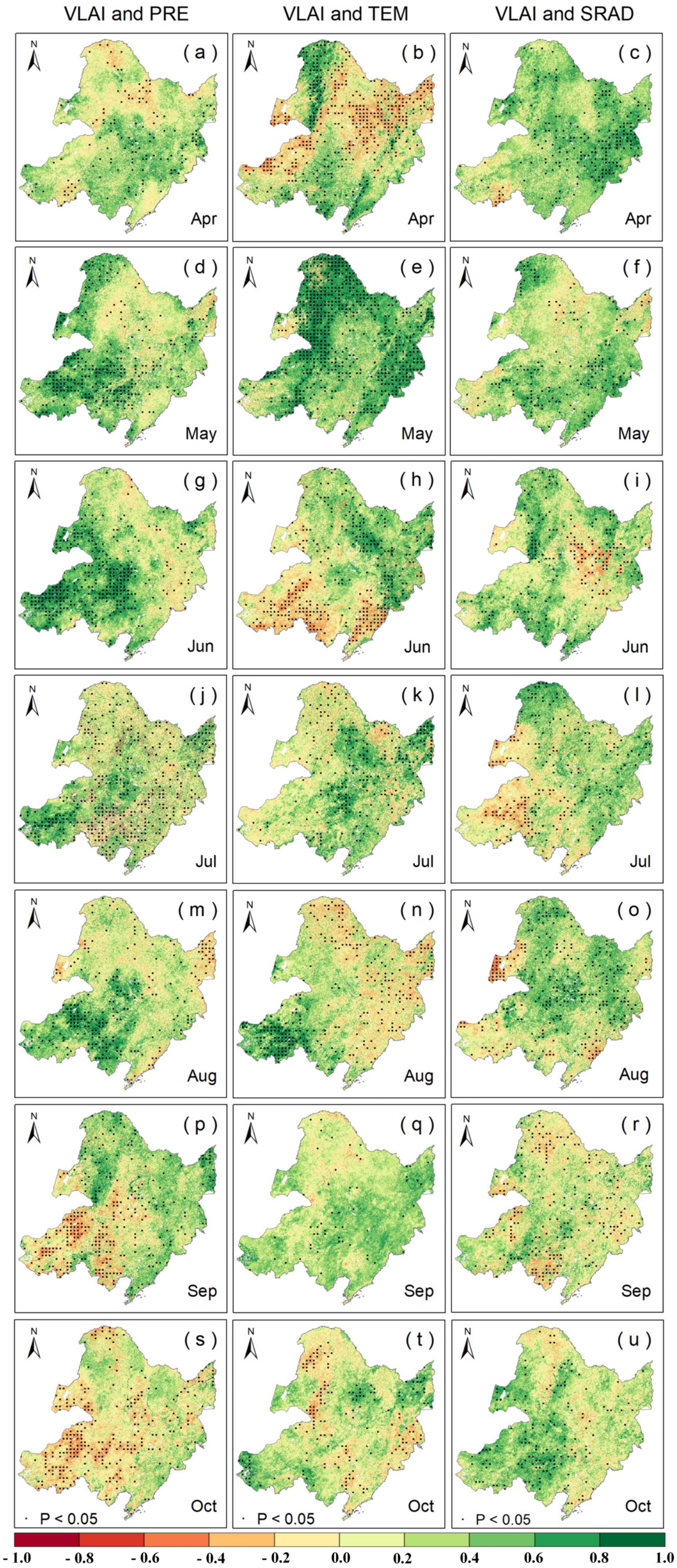

Figure. S11. Spatial distribution of partial correlations between VLAI and preseason climatic factors in Northeast China. The black squares indicate areas where VLAI is significantly affected by preseason climate factors(P<0.05).

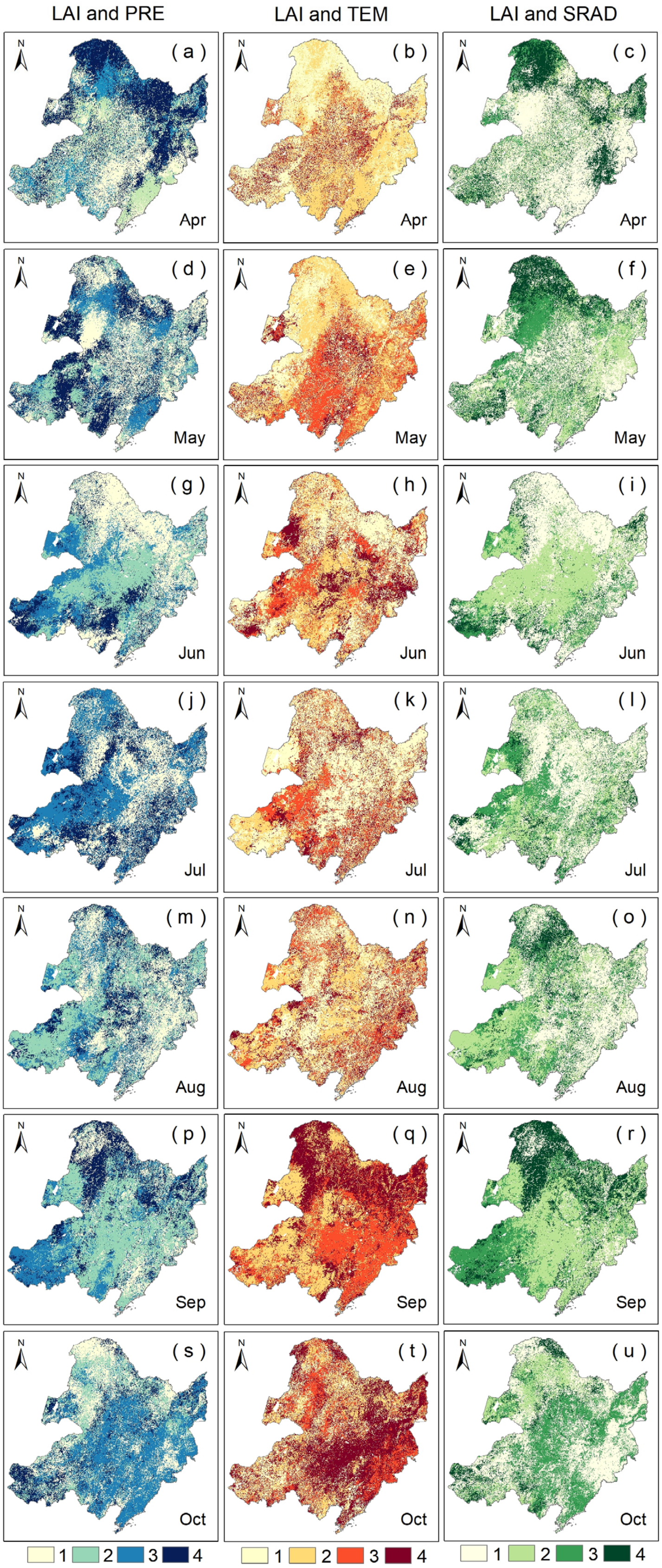

Figure. S12. Spatial distribution of preseason months for partial correlation analysis between LAI and climatic factors in Northeast China.

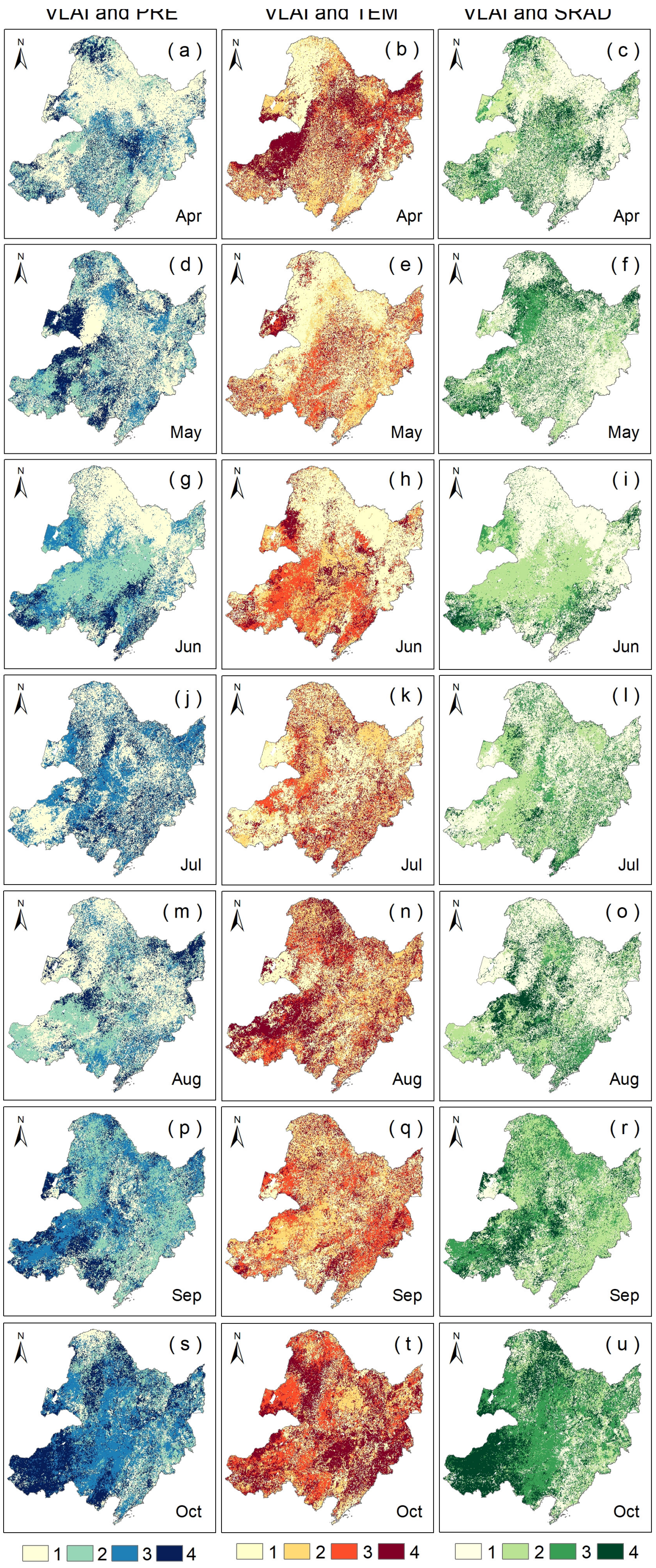

Figure. S13. Spatial distribution of preseason months for partial correlation analysis between VLAI and climatic factors in Northeast China.

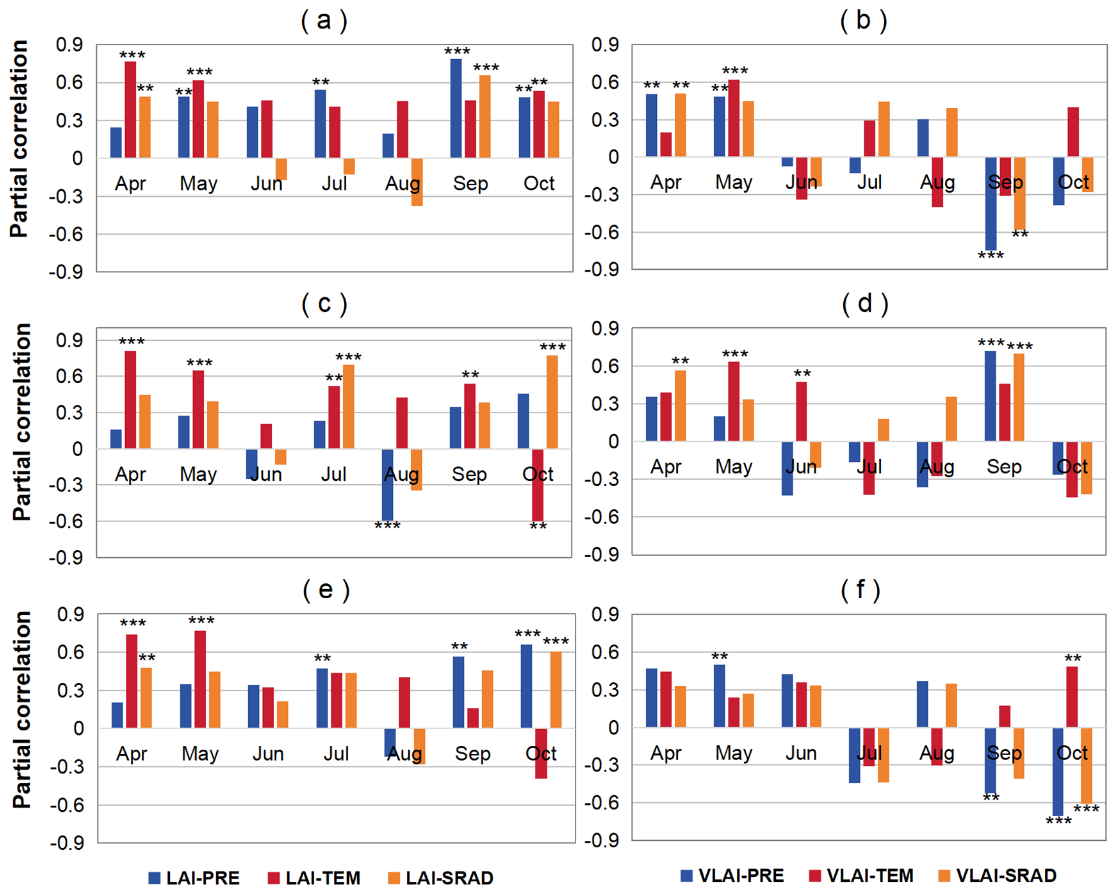

Figure. S14. Partial correlation statistical graphs between LAI and VLAI of grasslands (a and b), forests (c and d), and farmlands (e and f) and preseason climate factors in Northeast China. Significant correlations ($0.01 \le P < 0.05$) with climate factors are marked with "**", and highly significant correlations ($P < 0.01$) are marked with "***".
